# PEP for HIV prevention: are we missing opportunities to reduce new infections?

**DOI:** 10.1002/jia2.25942

**Published:** 2022-05-28

**Authors:** James Ayieko, Maya L. Petersen, Moses R. Kamya, Diane V. Havlir

**Affiliations:** ^1^ Kenya Medical Research Institute Center for Microbiology Research Kisumu Kenya; ^2^ School of Public Health University of California Berkeley California USA; ^3^ Makerere University College of Health Sciences and the Infectious Diseases Research Collaboration Kampala Uganda; ^4^ Division of HIV Infectious Disease and Global Medicine University of California San Francisco, San Francisco

1

There is newfound optimism for global efforts to reduce the estimated 1.7 million new HIV infections still occurring every year with expanded pre‐exposure prophylaxis (PrEP) access and newer biomedical prevention options on the horizon [1]. The increasing range of antiretroviral‐based prevention options, such as the dapivirine ring and long‐acting cabotegravir, move the concept of personal “choice” between biomedical HIV prevention options from the theoretical to the actual [2]. Post‐exposure prophylaxis (PEP) is not a new option for prevention in adults, but it is one that has been underutilized for decades in low‐ and middle‐income settings, largely being reserved only for high‐risk occupational exposures among healthcare workers. We challenge this approach.

The arguments to expand PEP access beyond occupational exposure are three‐fold. First, PEP is the only prevention option for adults that can be started after (vs. before) a high‐risk exposure. Based on human data and animal models, PEP works after needle stick or sexual exposure and needs to be started within 72 hours [3]. HIV risk exposures are often neither planned nor anticipated. Further, risk is dynamic and varies over time for each individual.

Second, PEP using currently recommended integrase strand transfer inhibitor‐based regimens is well‐tolerated and can be delivered outside of occupational exposure settings, including in settings, such as rural sub‐Saharan Africa (SSA), when operational barriers are addressed [4]. Estimated PEP efficacy is over 90%, which exceeds that of some other prevention options, such as the dapivirine ring [2]. PEP is particularly appealing due to the relatively short period of adherence required (i.e. for a 28‐day course). It effectively covers one‐time high‐risk exposures even among known high‐risk groups that do not have continuous ongoing risk, or those who opt to take a “break” from PrEP due to daily pill fatigue. When we offered PEP to persons with high‐risk sexual exposures in rural Kenya and Uganda using a patient‐centred approach that included offering services outside government clinics, 85% completed the 28‐day course and follow‐up testing. PEP was well tolerated, and there were no HIV seroconversions. Patients reported high satisfaction having an option that did not require ongoing daily medication [5].

Finally, PEP can be both a gateway to other prevention options for persons with ongoing exposure or a bridge for persons who have needs for short‐term protection, inclusive but not limited to occupational exposures [6]. Persons who access PEP have the opportunity to hear about other prevention options, such as PrEP. If they have ongoing risk, they can transition to PrEP with adherence support to overcome the challenge of taking a daily medication. We have found that some clients opt for repeated PEP and then make the self‐assessment that they would prefer to be on continuous PrEP only after this experience. We should both expect and embrace different on‐ramps and off‐ramps for HIV prevention options.

PEP can overcome some, but not all of the challenges of daily oral PrEP. Any daily oral pill started after a sexual encounter or needle sharing can be associated with stigma that can discourage the use of a preventive intervention even if the treatment requires only 28 days [7]. The similarity of PEP to HIV treatment can itself produce stigma of HIV infection that discourages uptake. Just like PrEP, persons can have side effects that make them unwilling to adhere to the regimen. All prevention options need to start with health literacy. Consumer education needs to expand beyond mere knowledge of options available and their side effects. It should include understanding patient goals and application of the knowledge in making choices of appropriate options to match their context [8].

If PEP requires no preplanning, is effective and requires only a short‐term 28‐day commitment and can potentially increase access to other biomedical options, such as PrEP, then why is it not more widely used? Guidelines per se are not the obstacle. In 2014, the World Health Organization expanded the recommendation for PEP for all persons who have occasional, unanticipated high‐risk exposure [9]. Drug supply also does not appear to be the major obstacle now that PEP regimens are the same as first‐line Anti‐Retroviral Therapy (ART) treatment regimens. Reasons for low uptake appear multi‐faceted and vary across settings. Despite being aware of PEP, men‐who‐have‐sex with‐men face barriers to access and use of PEP in Africa [10, 11]. Across multiple geographic contexts, healthcare providers are often unaware or have still not embraced the PEP option outside of the occupational setting or “key populations.” They may convey “judgemental” attitudes to clients seeking PrEP or PEP [8]. They may also view PEP for “emergency use” only, terminology used in the US Centers for Disease Control and Prevention guidelines that may inadvertently discourage PEP use [12].

What needs to happen to reduce barriers to PEP? As countries update HIV prevention guidelines with availability of new products, there needs to be renewed focus on maximizing entry points into HIV prevention. Providers need refresher trainings on prevention that include PEP, and its potential role as an on‐ramp or as a bridge to other options. Trainings need to emphasize the important role of providers to reduce stigma with PEP and to resist the temptation to decide what they think is best option for the client. Provision of the full 28‐day course of PEP at a single clinical visit, with ongoing remote adherence counselling, can reduce transport and cost barriers to the client [13, 14]. PEP availability needs to be expanded to locations outside the clinics, such as through community‐based retail drug shops [15]. Community outreach needs to empower clients to increase consumer demand and PEP delivery systems that meet their needs. Researchers need to evaluate even shorter PEP regimens.

In conclusion, a combination approach to preventing HIV infection, including behavioural, biomedical and structural approaches tailored to those at greatest need, must be adapted to realize the effectiveness of different HIV prevention options and reduce new infections [16]. Collectively, including more options can enhance the success of the overall prevention armamentarium. PEP is an efficacious HIV prevention option that has largely been underutilized. This defines a missed opportunity to prevent HIV infection associated with high‐risk exposures in high‐burden disease settings. A shift in thinking around the use of PEP by policy makers and implementing programs is needed as we move to one of the most exciting eras in HIV prevention.

## COMPETING INTERESTS

All authors research support from NIH. DVH received non‐financial support from Gilead Sciences to her institution for research studies.

## AUTHORS’ CONTRIBUTIONS

JA was the lead author and compiled the first draft. DVH, MLP and MRK reviewed and edited the manuscript. All authors read and approved the content of this Viewpoint.

## DISCLAIMER

The contents of the Viewpoint represent the views of the authors and not necessarily the views of their affiliated institutions.
